# The Science Versus the Art of Veterinary Surgery1Read before the New York State Veterinary Medical Society, Hotel Metropole, New York City, September 15, 1898.

**Published:** 1898-10

**Authors:** Robert W. Ellis

**Affiliations:** Lecturer on Obstetrics, American Veterinary College, New York


### THE SCIENCE *VERSUS* THE ART OF VETERINARY SURGERY.[1]

By Robert W. Ellis, D.V.S.,
LECTURER ON OBSTETRICS, AMERICAN VETERINARY COLLEGE, NEW YORK.

Essay-writing is not my strong point, so upon being requested to present something at this meeting I have jotted down a few thoughts as they have presented themselves to me, under the imposing title of "The Science *versus* the Art of Veterinary Surgery."

In looking over the field of surgery, with its advances in the various operations for the alleviation of pathological conditions, as well as for other advantages to be derived from them, the question has presented itself to me, Is the art of surgery keeping abreast with the science in general practice? And in looking about me for an answer to the question, my observations have all led me to believe that it is not.

Among the advances we note the introduction of new operations for the alleviation of pathological conditions which were incurable previous to their introduction, as arytenectomy for roaring due to laryngeal hemiplegia, and the still more recent arytenoideraphy for the alleviation of the same condition. And for chronic tendonitis, periostosis of the fetlock and like conditions that resist firing, blistering, and other modes of treatment, there is relief to be obtained by mesoneurectomy and median neurotomy. And besides the new operations that are continually being presented there are new and advanced methods of performing old operations made possible by strict asepsis.

One circumstance which leads me to this unwilling conclusion is that there are comparatively few men in general practice who ever attempt any of the classical operations, and not a few who evade the more ordinary ones, even if their results by other modes of treatment are not so beneficial. Yet I have heard some of those same men earnestly proclaiming to some interested listener the great strides that have been made in veterinary surgery, and telling of the different new operations now in vogue in a manner sufficiently convincing that they were not at all behind in the literature of the day on these subjects, although entirely wanting in the application of the methods they were describing, having never performed them themselves, but contentedly basking in the reflection of the glory of those members of the profession who had. Such

[1] Read before the New York State Veterinary Medical Society, Hotel Metropole, New York City, September 15, 1898.

content, gentlemen, and the feeling that "such operations are all right for Prof. So and So, but are too much for me" is the secret to a great extent of the art of surgery not keeping abreast with the science in general practice. And should each one of us ask ourselves the question, "Am I up to my ideal veterinary operator, or am I on the way to getting up to my ideal?" or, on the other hand, "Am I not possibly behind some of the bold, though perhaps less scientific operators of a decade ago?" which of the two questions, gentlemen, would many of us have to answer in the affirmative? I fear the latter.

Now, the next question that would naturally present itself is, "Why is this so?"

I think to some extent (but to how great an extent I am not prepared to say) the lack of inclination or adaptability to that particular branch of practice which soon results in the feeling of "content" before alluded to. But I believe another and, perhaps, the principal reason is the lack of a sufficient number of the variety of cases in general practice to attain proficiency in some of the more delicate and classical operations. Another reason is that every veterinarian is not fortunate enough in the beginning to have facilities, such as suitable places to operate, etc., or he, at least, feels that he has not, coming as he does just fresh from college, where he has been accustomed to seeing operations performed in spacious operating-rooms with all the necessary appointments, and he is very apt to send the case to some one that has, expecting to be better prepared later on. If he is in the vicinity of a veterinary college he sends the patient there, and thus it is that you will observe house-surgeons in college hospitals almost invariably become good operators by being constantly brought in contact with that kind of work. In other words, a fellow-graduate of the house-surgeon goes out to build up a practice, just as well prepared for his work as the house-surgeon; but finally, when he meets a case that requires some special operation, he reflects that he has not the facilities yet to handle that case, and so sends it to the college hospital. That is an operation lost to him and gained by the house-surgeon, and in that way he runs back while his classmate goes ahead. In other words, the art of surgery with him has not kept abreast with the science, for during all his spare moments he has been reading the current literature of the day, possibly has bought a new book or two on surgery, and has learned with much interest of one or two new operations that have come into veterinary surgery since he graduated, all of which have undoubtedly been performed at the veterinary schools since that time, also.

These remarks, gentlemen, remember, apply principally to classical operations of the nature of those mentioned in the beginning of my paper, although many of the operations performed by some of us in our everyday work, such as neurotomy, both median and plantar, bitch-spaying, castration of the male, to say nothing of the more common operations of firing, etc., are entirely evaded by some.

Assuming, then, that I am right in stating that the art of surgery does not keep abreast with the science in general practice, you will admit that this is not as it should be. That point being admitted, a remedy of the condition is, perhaps, not out of place for consideration in this paper.

In reference to the first class of practitioners mentioned, viz., those entirely disinclined and consequently unsuited for that particular branch of practice, nothing is to be said, as they have a perfect right to leave that branch for those so inclined if they wish to do so; but for practitioners in general, operating more or less in minor surgery, but who have never attempted the more intricate and, perhaps, we might add, more recent operations, I believe that a realization on their part that the principles of surgery are the same no matter in what class the operation may rank, and that in the higher-class operations simply an application of the same rules and principles is required as in more simple ones (except that, perhaps, more care and skill are required in their application) will tend strongly toward removing the imaginary gulf between the two classes. So that to prepare for a new and, perhaps, difficult operation is simply to make a careful review of the regional anatomy, study the steps of the operation carefully from the literature on it, get the details as to what instruments, etc., are required for the particular operation in question, and then proceed carefully, yet boldly and confidently, knowing that you have the details all in your mind, and feeling that as a surgeon you are capable of carrying them out; and, as a rule, success will crown your efforts.

Again, for those who do not feel disposed to undergo the mental strain of performing a delicate operation for the first time upon a valuable animal belonging, perhaps, to a valuable client, but who would prefer entering upon it with that ease and confidence born of familiarity, veterinary surgery offers us the advantage of being able to procure a valueless subject for experimentation, and in the more delicate operations, especially if a man has never performed any in that class, I think this plan advisable; for, although he would probably perform the operation all right the first time if he had given the proper amount of attention to the details of it, regional anatomy, etc., by way of preparation, he would certainly perform it better, or perhaps I should say easier, the second time. The presence, in these experimental operations, of a practitioner familiar with it will materially aid in getting the details readily.

The extended day sessions of our national association render it possible to have practical operative surgery as a part of the programme, which is, of course, incompatible with the short evening sessions of our local associations, meeting monthly, quarterly, etc., to say nothing of the indisposition of the members to entering into such a programme after their routine of work all day. They more naturally, after their dinner, prefer sitting in a comfortable meeting-room and listening to a paper or discussion on some interesting subject by fellow-members or themselves entertaining by the same means. And at these meetings, if occasional papers were read by members, describing their particular *modus operandi* in certain operations performed by them, they would, with the discussions that would naturally follow, prove extremely interesting and instructive, and tend to improve our methods of operating by a healthy exchange of ideas (you are all familiar with the evils of in-and-in-breeding), for while we are cognizant of the benefits to be derived from scientific intercourse, which we all concede tends to advance us, we must see to it that we advance symmetrically; for, like growing plants, we have a tendency to lean toward the side upon which the sun shines most, and I feel certain that papers on operative surgery are painfully in the minority, at least in our local association meetings. I am sure that there are not a few of us who would, at least, like to become more familiar with such operations as arytenoideraphy, arytenectomy, median neurotomy, castration of cryptorchids, caponizing, amputation of the penis, extirpation of the clitoris, and ovariotomy in mares afflicted with nymphomania, and, perhaps, many others which I could mention, were you not all familiar with them, and papers of that order would, I think, act as a stimulus for us to perform more of the higher-class operations either therapeutically or experimentally, and thereby tend to advance the art of surgery equally with the science in general practice.

To sum up, then, I have arrived at the following conclusions:

1. That the art of surgery has not kept pace with the science in general practice.

2. That such is the case through a failure on the part of practitioners to realize that the principles of surgery are the same in all classes of operations.

3. That said principles can be applied in operations of any class by graduates in veterinary surgery by a careful study of the details pertaining to each particular operation.

4. That every practitioner owes it, as a duty to himself and his clients, to keep himself up to date in the art of surgery, just as much as in the use of new drugs for the relief of pathological conditions, so that he may be prepared at any time to perform any operation that may present itself to him.

5. That there are comparatively few papers pertaining to operative surgery presented at our association meetings; and

6. That an increase in such papers would produce an increase of interest and a desire to perform many operations previously considered beyond us, as a result; and the problem of how to advance the art of surgery with the science thereby solved.

Now, gentlemen, while I realize that my paper has not done justice to its title and that I have, at best, made but a superficial survey of the subject which I have attempted to cover, I hope that it will at least suffice to elicit the valuable opinions of some of my colleagues present that we may all profit by them.

